# Three-dimensional single molecule localization close to the coverslip: a comparison of methods exploiting supercritical angle fluorescence

**DOI:** 10.1364/BOE.413018

**Published:** 2021-01-12

**Authors:** Philipp Zelger, Lisa Bodner, Martin Offterdinger, Lukas Velas, Gerhard J. Schütz, Alexander Jesacher

**Affiliations:** 1 Division for Biomedical Physics, Medical University of Innsbruck, Müllerstraße 44, 6020 Innsbruck, Austria; 2 Division of Neurobiochemistry, Biooptics, Medical University of Innsbruck, Innrain 80–82, 6020 Innsbruck, Austria; 3 Institute of Applied Physics, TU Wien, Getreidemarkt 9, 1060 Vienna, Austria

## Abstract

The precise spatial localization of single molecules in three dimensions is an important basis for single molecule localization microscopy (SMLM) and tracking. At distances up to a few hundred nanometers from the coverslip, evanescent wave coupling into the glass, also known as supercritical angle fluorescence (SAF), can strongly improve the axial precision, thus facilitating almost isotropic localization performance. Specific detection systems, introduced as *Supercritical angle localization microscopy* (SALM) or *Direct optical nanoscopy with axially localized detection* (DONALD), have been developed to exploit SAF in modified two-channel imaging schemes. Recently, our group has shown that off-focus microscopy, i.e., imaging at an intentional slight defocus, can perform equally well, but uses only a single detection arm. Here we compare SALM, off-focus imaging and the most commonly used 3D SMLM techniques, namely cylindrical lens and biplane imaging, regarding 3D localization in close proximity to the coverslip. We show that all methods gain from SAF, which leaves a high detection NA as the only major key requirement to unlock the SAF benefit. We find parameter settings for cylindrical lens and biplane imaging for highest z-precision. Further, we compare the methods in view of robustness to aberrations, fixed dipole emission and double-emitter events. We show that biplane imaging provides the best overall performance and support our findings by DNA-PAINT experiments on DNA-nanoruler samples. Our study sheds light on the effects of SAF for SMLM and is helpful for researchers who plan to employ localization-based 3D nanoscopy close to the coverslip.

## 1. Introduction

Single molecule localization microscopy (SMLM) is a powerful microscopy technique for obtaining spatial resolutions far below the diffraction limit [1–3]. Localization along the axial direction (z-axis) poses a particular challenge for microscopes, because the depth of focus is – even for highest numerical apertures – significantly larger than the lateral width of a molecule image. The consequence is an imbalance between lateral and axial localization precisions, with the latter one being noticeable worse.

This issue has been tackled by several research groups in the past decade and numerous solutions to the problem exist (see [4,5] for recent reviews on the topic). Most of these methods foresee suitable PSF modifications, i.e., they alter the way a point-source is imaged onto the detector. Examples to this end are cylindrical lens imaging [6], 4-Pi detection [7], multi-plane imaging [8,9] or more advanced methods, which make use of specifically designed optical elements or diffractive patterns displayed on spatial light modulators [10]. These elements are typically placed in a Fourier plane, i.e., an optical plane that is conjugated to the back focal plane of the objective lens. Examples are imaging with helical [11–13], tetrapod [14], or self-bending [15] PSFs, parallax imaging [16] or the use of a phase ramp in the back focal plane [17], which is related to parallax imaging.

Some techniques do not physically alter the optical transfer function, but measure additional information contained in the PSF of a regular microscope. Examples are phase-sensitive imaging [18], photometry [19] or lifetime measurements [20].

Finally, there exist methods which exploit information that is only available if the sample lies at distances smaller than a few hundred nanometers from the glass / buffer interface, or even less. In spite of this small working range, there exist numerous important applications for SMLM in this regime, for instance the imaging of cellular membranes [21–23], bacteria [24,25], viruses [26,27] or applications in material science [28]. Some members of this method family are exploiting metallic [20] or combined metallic-dielectric [29] coverslip coatings, which are known to shorten fluorescence lifetimes and to enhance the signal.

Other methods known as SALM [30], respectively DONALD [31] or the recently introduced direct SALM (dSALM) method [32] draw information from supercritical angle fluorescence (SAF), i.e., the part of the non-propagating near field turning into propagating waves inside the coverslip whenever an emitter is sufficiently close. To measure SAF, SALM and dSALM employ beam splitting and two-channel detection.

Recently, we have shown that the SAF advantage can also be exploited with a regular, unmodified microscope [33]. If the objective lens is moved towards the sample by about 500 nm, the axial localization precision is significantly improved, much stronger compared to imaging under similar conditions inside the bulk of the buffer. The resulting performance is comparable to that of SALM, if SALM is used in conjunction with optimal data processing [33].

Our previous work highlighted the fact that SAF intrinsically improves any localization method if the objective NA is larger than the refractive index of the buffer medium. Therefore, it appears worth revisiting existing methods and investigating their performance close to the coverslip, taking SAF emission into account. Although different localization methods have been regularly benchmarked in the past [4,17,34–36], we find that the main focus of these studies lies on bulk imaging. SAF light is often not considered.

In this paper, we investigate the performances of some of the most popular 3D localization methods at distances of up to λ/3 to the coverslip, where evanescent field coupling plays a role. At a distance of λ/2, the SAF energy plays no significant role any more. It’s ratio to the so-called under-critical angle fluorescence (UAF) energy is merely on the order of 10%.

Our comparative study includes off-focus [33,37], biplane [8,9] and astigmatic imaging [6] as well as SALM [30,31]. Further, we briefly discuss the only recently experimentally demonstrated method dSALM [30], which promises to generally outperform SALM.

Localization precision values are doubtlessly very important, but do not draw a full picture of a method’s qualities. Aspects of sensitivity to aberrations, multi-emitter events or anisotropic fluorescence emission caused by a hampered rotational diffusion of fluorophores are likewise important, but often neglected. These effects lead to biased results, which are only noticeable if the sample structure is known a priori, for instance when using specific calibration samples [38–40]. Therefore, we also compare SMLM methods in view of robustness to the most relevant phase aberrations, dipole effects and multi-emitter events. Related studies exist for individual influence factors and methods [41–43], but a direct comparison such as presented here has not been published to the best of our knowledge. We note that various methods have been developed which estimate dipole orientations as well (see Ref. [44] for a review of methods).

Here we use a common mathematical model M to describe the numbers of photons detected in each camera pixel as a function of five parameters: The molecule’s 3D position (x,y,z), the number of photons emitted by the molecule and detected by the camera σ and the background fluorescence level β: M(x,y,z,σ,β)=β+σ⋅h(x,y,z). Here, h is the three-dimensional PSF, which in our case represents the image of an isotropic point-emitter, e.g. a fast tumbling fluorescent molecule. Depending on the particular method, h can be either the PSF of an unmodified objective pupil, an astigmatic PSF or even a split PSF as in the case of SALM or biplane imaging.

## 2. Localization performance close to the coverslip

When imaging deeper inside of a live biological sample, it is common to use water immersion lenses, as the refractive indices of water and sample are similar and spherical aberrations minimized. Conversely, when imaging close to the coverslip, spherical aberrations are almost negligible and it is advised to use oil objectives with higher numerical apertures for the sake of SAF collection.

For all simulations presented in this paper, unless otherwise stated, we use a specific set of experimental parameters, which are listed in the following: NA = 1.49 (immersion oil and coverglass RI = 1.518), emission wavelength $\lambda_{em}$ = 670 nm, effective pixel size = 100 nm, camera readout noise = 1 electron RMS per pixel, camera dark noise = 0, quantum efficiency = 75%, axial working range = 220 nm ($\approx \lambda_{em}/3$), buffer medium = water. The PSF is modelled according to Ref. [45].

**Fig. 1.**
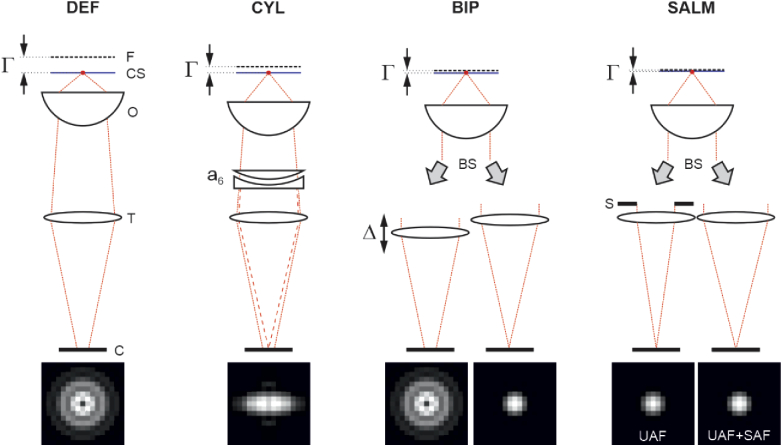
**Sketch of compared methods**; F⋯focal plane, ⋯coverslip, O⋯objective lens, T⋯tube lens, C⋯camera, ⋯AF-blocking aperture, BS⋯beam splitter;

We investigate the following 3D SMLM methods: A) defocused imaging (DEF), B) cylindrical lens imaging (CYL), C) biplane imaging (BIP) and D) SALM. Every method can be tuned via specific parameters: DEF and SALM have only one parameter, the off-focus value Γ [33], which is defined as the distance the objective is displaced towards the sample. Γ=0 means that the coverslip/buffer interface is in focus. Γ is the actual value to be set on the microscope z-stage and does not depend on the refractive index of the buffer medium. CYL and BIP imaging have two parameters each: BIP has a defocus parameter Γ as well as a further parameter Δ, which is defined as the difference between the defocus values of both imaging channels: $\Delta =\Gamma_{2}-\Gamma_{1}$. CYL is defined by one defocus parameter Γ and an astigmatism coefficient $a_{6}$. The index number is explained by the fact that astigmatism is modelled by first order Zernike astigmatism $Z_{6}$ (according to the Noll indexing scheme [46]) in the objective pupil. A pair of convex/concave cylindrical lenses, one being rotatable, is used in our experiments to introduce astigmatism. Sketches of the respective setups are shown in Fig. 1, including specific elements such as the cylindrical lens pair to set astigmatism in CYL, the movable tube lens to set Δ in BIP or the SAF-block used in SALM. The bottom of the figure shows simulated images of a molecule sitting at the coverslip (z=0) for each method. Note that the effect of refraction between the coverslip and water has been neglected in the sketch to facilitate a clearer visualization. In reality, the physical distance between focal plane and coverslip/water interface is not identical to Γ.

**Fig. 2.**
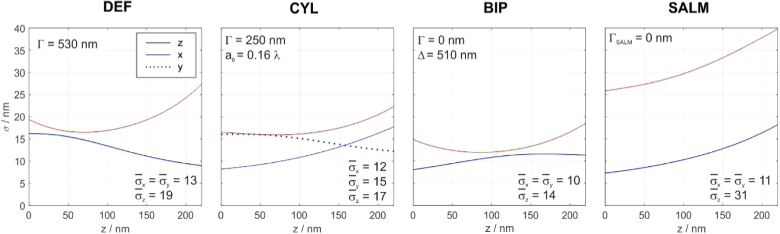
**Comparison of localization precisions**; Parameter settings are chosen for optimal z-localization. Biplane imaging shows the best overall performance. The mean precision values (in nm) are stated below the curves. Boundary conditions: UAF signal = 2000 photons, background level = 100 photons per pixel, NA = 1.49.

The parameters specific to each method have been optimized with Matlab to provide the best axial precision over the considered z working range of $\lambda_{em}/3$. The graphs in Fig. 2 show 3D localization precisions for molecule positions ranging from 0 to 230 nm. The graphs are based on calculated Cramér-Rao lower bounds [33,47,48], assuming a UAF signal of 2000 photons and a background level of 100 photons per pixel. The best parameters obtained are stated above the graphs. An investigation on how the localization precision suffers from wrongly set parameters is contained in the appendix (section 7.1).

Referring the calculations to a constant UAF signal rather to a constant total signal (i.e. UAF + SAF) provides more realistic curves for fluorophores with low quantum yield, where UAF and SAF signals are not competing [32]. For high quantum yields, the onset of SAF will somewhat decrease UAF, but the resulting changes to the CRLB curves are be minor.

Apparently, biplane imaging performs best. For the assumed signal and background level, the average z-precision value of BIP is about 30% / 20% / 50% lower than for DEF / CYL / SALM. At the same time, the transverse precision values are about 20% lower than for DEF and CYL and approximately matching that of SALM. This general superiority of BIP is valid over a large signal-to-background ratio range. Localization precisions for various signals and background levels for each method, assuming the respective optimal imaging parameters, are presented in the appendix (section 7.2). We further find that the ideal parameters vary little over a wide range of signal and background levels. For instance, the ideal biplane settings are (Γ,Δ)=(0, 570 nm) for (σ,β) = (50.000 photons, 0) and (Γ,Δ)=(0, 490 nm) for (σ,β) = (500 photons, 100 photons/pixel). We note that the performance data presented in Fig. 2 are independent from the excitation method. TIRF excitation for instance will lead to a reduced background, but has otherwise no influence on the curve characteristics.

In addition, we have performed simulations on the only recently experimentally demonstrated method dSALM, where SAF and UAF contributions are separately imaged without blocking any light [32]. Since the performance of dSALM depends strongly on the background levels of the two imaging channels and thus also on the illumination conditions (TIRF vs. epi-fluorescence excitation at low angles), we refrain from including the method in Fig. 2. However, we provide additional information about the performance of dSALM in the appendix.

The optimal imaging parameters depend on experimental boundary conditions. Their approximate dependence on peak emission wavelength and maximal axial working range are shown in Table 1. These estimates have been found from a series of simulations considering many different parameter combinations and are useful over a broad range of commonly used effective pixel sizes and signal to background ratios. Specifically, all parameter combinations of the following sets have been tested: signal = [500, 5000] photons; background = [20, 200] photons per pixel; wavelength = [500, 600, 700] nm; maximal z-range = [70, 140, 210] nm; camera pixel size = [6.5, 13] µm. However, they are only valid in the SAF-effective range, that is up to about $\lambda_{em}/3$, an NA of 1.49 and a buffer refractive index of 1.33. The optimal defocus value of SALM is always close to zero.

**Table 1.** **Imaging parameters for optimal z-precision**; The parameters are given as functions of the peak emission wavelength and maximal axial working range.

	Γ	$a_{6}/\lambda \text{RMS}$	Δ
DEF	0.50 $\lambda_{em}$ + 0.80 $z_{max}$	n.a.	n.a.
CYL	0.15 $\lambda_{em}$ + 0.60 $z_{max}$	0.17	n.a.
BIP	−0.25 $\lambda_{em}$ + 0.80 $z_{max}$	n.a.	0.70 $\lambda_{em}$
SALM	0	n.a.	n.a.

## 3. Impact of aberrations

Objective lenses are designed for aberration free imaging at the coverslip. However, this applies only to the central part of the field of view (FOV). At distances of some tens of microns from the optical axis, field aberrations such as astigmatism and coma become noticeable. Furthermore, spherical aberrations of magnitudes below the diffraction limit as defined by the Maréchal criterion [49,50] (72 mλ) are often present. Such aberrations would be barely visible in widefield imaging, but can have a noticeable effect on both precision and accuracy in SMLM as we will show in the following. Some objective lenses feature correction collars, which allow for compensating spherical aberrations. However, we have noticed that the ideal, aberration-free collar setting can be somewhat off the supposedly optimal position (i.e. 0.17 if standardized, high precision coverslips of 170 µm thickness are used) and that setting the collar to this value can introduce spherical aberrations which are significantly larger than the Maréchal threshold.

**Fig. 3.**
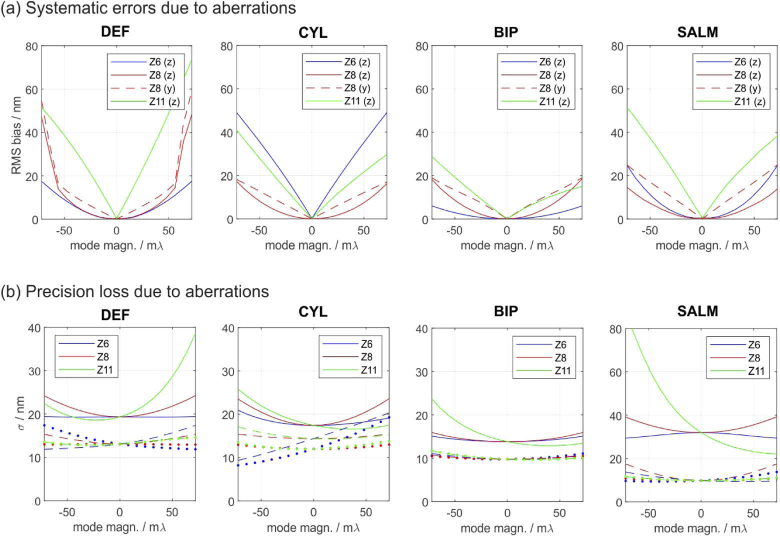
**Systematic errors and precision losses caused by phase aberrations.** Each method is applied at it’s respective ideal parameters shown in Fig. 2. The effects of astigmatism (Z6), coma (Z8) and spherical aberrations (Z11) are shown. Axial biases and precisions are marked by solid lines, lateral ones by dashed/dotted lines. (a) Systematic errors: astigmatism and spherical aberrations only affect the z-estimates; coma also affects one lateral coordinate. BIP performs best. (b) Precisions: SALM and off-focus imaging suffer most from spherical aberrations. Again, BIP shows the best performance. Simulation parameters: NA=1.49, $\lambda_{em}$ = 670 nm, $z_{max}=\lambda_{em}$/3, eff. pixel size = 100 nm. UAF signal = 2000 photons, bg-level = 100 photons / pixel. For (a), the images were assumed to be noise-free (no shot noise or camera noise).

In the following, we investigate the impact of astigmatism, coma and spherical aberrations on the accuracy and precision of different SMLM techniques as well as their capability to remove aberrant molecule fits from the data using log-likelihood ratio (LLR) tests. The aberrations are modelled in the objective pupil via the 1st order Zernike terms $Z_{6}$, $Z_{8}$ and $Z_{11}$ according to the Noll indexing scheme [46]. The four methods under concern (DEF, CYL, BIP, SALM) are applied at their respective ideal parameter settings shown in Fig. 2. To obtain the results shown in Fig. 3(a), aberration-afflicted but noise-free images of molecules at different z-positions have been calculated and evaluated under the assumption of an aberration-free PSF model [45]. The figure shows mean systematic position errors (biases) introduced by phase aberrations ranging from −72mλ to +72mλ. The plotted bias curves represent RMS values over the entire z-range from zero to $\lambda_{em}/3$, therefore they are necessarily positive.

The following conclusions can be drawn from Fig. 3(a): a) Even small aberrations below the diffraction limit can cause relatively large systematic errors on the order of 20 to 60 nm, which is a significant fraction of the z-working range. This circumstance highlights the importance of carefully characterizing the PSF, for instance using phase retrieval, or using adaptive optics to compensate for them. b) Methods are especially vulnerable to aberrations which directly affect their sources of information: CYL for instance is particularly sensitive to astigmatism (Z6) and DEF to spherical aberrations (Z11), which are related to defocus. c) BIP shows overall the highest robustness, which is presumably due to the diversity provided in the two recorded images.

The impact of aberrations on the localization precisions are visualized in Fig. 3(b). Here it is assumed that the aberrated PSF is known and used for data evaluation. The biases shown in (a) hence drop to zero. However, it is important to note that a loss of precision cannot be regained using purely numerical methods such as an adapted PSF model, but only by physically correcting the PSF using adaptive optics.

The following conclusions can be drawn from Fig. 3(b): a) DEF and SALM are strongly affected by spherical aberrations. At an aberration magnitude of only $Z_{11}$=72 mλ, the threshold to the diffraction limit, the z-precisions already drop by a factor of two (DEF) or even three (SALM). Interestingly, SALM can benefit from small positive spherical aberrations. b) BIP is the most robust amongst the investigated methods. Especially the lateral localization precisions are barely affected by weak aberrations.

The robustness of a method to aberrations is an important characteristic. However, of comparable importance is a method’s ability to identify them in the first place, such that afflicted localizations can be removed from the data. To some extend this is possible by quantifying the discrepancy of the molecule image to the best model fit, for instance by calculating the log-likelihood ratio (see appendix 7.4 for definition and details). The higher the LLR, the larger the remaining fit discrepancy and the easier it is for the algorithm to identify problematic localizations and discard them. Conversely, a small LLR means that the aberration has only a small impact on the PSF shape or the induced changes are similar to those caused by variations of the parameters of interest, i.e., x, y, z, σ, β.

**Fig. 4.**
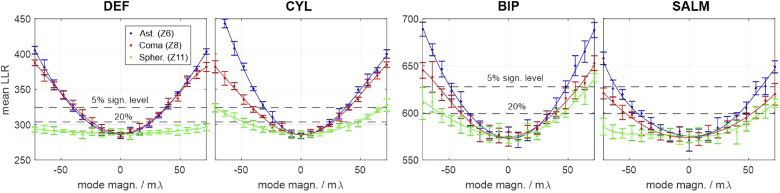
**Log-likelihood ratios of molecule image fits for varying aberration magnitudes.** The plots quantify each methods’ ability to reveal the presence of aberrations. Every data point represents an average LLR value over the entire z-range. Each errorbar marks the standard deviation of the respective z-averaged LLR value, calculated from 5 subsequent trials, assuming the signal and background values stated below. The black dashed lines mark the 5% and 20% significance thresholds. If the curves fall below these lines, the aberration remains undetected by the localization algorithm, thus corrupting the data by introducing the biases shown in Fig. 3. Assumed parameters: UAF signal = 5000 photons, background = 100 photons per pixel.

Figure 4 visualizes aberration induced LLR changes for all methods. Each data point represents an averaged LLR value of 23 simulated molecule images equally spread over the entire z-range. Each image contains 5000 signal photons and a background level of 100 photons per pixel. Parabolic lines have been fitted to each set of data points to guide the eye. The horizontal black dashed lines mark the 5% and 20% significance levels. This means that there is a 5% / 20% chance that the LLR value of an aberration free molecule image will lie above the respective levels. Apparently, if the 5% level is chosen as a rejection threshold, none of the methods is capable of filtering out spherically aberrant molecule images, if the aberration magnitude is below the diffraction limit, even at the assumed high signal level. For DEF and SALM this holds even true for the less strict 20% significance level.

The following conclusions can be drawn from Fig. 4: a) The presence of aberrations below the diffraction limit is only detectable at very high signal to background levels. b) Field-dependent aberrations (astigmatism, coma) are most easily revealed by DEF and CYL. c) Spherical aberrations are generally harder to detect, especially by DEF and SALM. This is especially unfavourable, given the high systematic errors and precision losses they cause for these methods.

## 4. Systematic errors caused by fixed dipole orientations

In many cases, fluorescent molecules can be assumed as “quick tumblers”, which means that their emission dipoles homogeneously explore the entire spatial angular space within one image exposure. This has also motivated our choice to model the PSF as image of an isotropic emitter, i.e., the sum of x-, y- and z-dipole intensity images of equal magnitudes. However, this isotropy can be broken, for instance if the excitation polarization shows an orientational preference and a mounting medium of high viscosity is used, which slows down the rotational diffusion such that its characteristic time scale becomes comparable to the fluorescence lifetime [51]. There are also cases where the freedom of dipoles to rotate is partially hampered or even fully frustrated [44]. In such cases, the model of an isotropic emitter is ill-suited and systematic localization errors occur.

One solution to this problem is the removal of the radial polarization component of the collected fluorescence in the back focal plane [52,53], which has been identified to be the cause of the anisotropy. This however, comes at the cost of a reduced signal and thus localization precision. More recently, polarized two-channel imaging with separated x-y localization has been proposed to mitigate the problem [54]. Furthermore, recent advances have been made towards the joint estimation of position, orientation and wobbling parameters using polarized four channel detection [55–57].

However, in experiments where the dipole orientation is not of interest and the conditions allow for “quick tumblers”, it is sufficient to use a simpler measurement strategy, e.g. one of those compared in this study. Even so, a certain robustness against potential violations of the emission isotropy appears to be a beneficial property.

To this end, we investigate the “worst case scenario”, that is estimating the 3D position of a fixed emission dipole using an isotropic emitter model, because the occurring systematic errors mark “extremal values” and any realistic bias is likely to be smaller. For symmetry reasons it is sufficient to simulate images of dipoles that vary only by $\theta_{dip}$, i.e., the including angle between dipole and optical axis. The azimuthal dipole angle $\Phi_{dip}$ is set to zero, except for the method CYL, which is sensitive to variations of $\Phi_{dip}$ due to its own intrinsic anisotropy established by the cylindrical lens. For CYL, two azimuthal angles, $\Phi_{dip}$ = 0 and $\Phi_{dip}$ = $90^{\circ }$ are considered.

Noise-free images of dipoles with θ varying between 0 and $90^{\circ }$ and various z-positions have been simulated and their 3D positions estimated using the isotropic emitter model and the four methods under concern. For the two-channel methods, the lateral molecule coordinate estimates $\hat{x},\hat{y}$ are calculated as weighted averages of the respective position estimates from the two images (${\hat{x}}_{1},{\hat{y}}_{1},{\hat{x}}_{2},{\hat{y}}_{2}$), with CRLB-based variances used as weights:

(1) $$\hat{x}=\frac{\sigma_{x1}^{-2}{\hat{x}}_{1}+\sigma_{x2}^{-2}{\hat{x}}_{2}}{\sigma_{x1}^{-2}+\sigma_{x2}^{-2}},\;\;\hat{y}=\frac{\sigma_{y1}^{-2}{\hat{y}}_{1}+\sigma_{y2}^{-2}{\hat{y}}_{2}}{\sigma_{y1}^{-2}+\sigma_{y2}^{-2}},$$

**Fig. 5.**
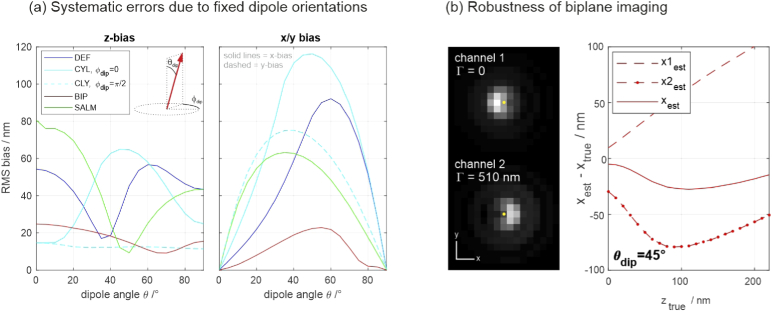
**(a) Systematic errors of axial and lateral position estimates**, caused by imaging a fixed dipole with orientation angle $\theta_{dip}$ in combination with using an isotropic emitter model. **(b) Robustness of biplane imaging to dipole orientations.** The images show a $\theta_{dip}$=$45^{\circ }$ tilted dipole in both channels. The yellow dots mark the true dipole position. Apparently, the biases have opposite directions, largely compensating the net bias. This is also supported by the plot on the right, which shows the x position estimates of the two channels as well as the joint estimate for the $\theta_{dip}$=$45^{\circ }$ dipole.

The RMS values of the resulting positional errors over the entire z-range are plotted in Fig. 5. The probably most interesting result is the surprising robustness of biplane imaging, which remains mostly below 20 nm, even for the severe model mismatch considered in this simulation. A closer investigation reveals that the robustness of the lateral estimate is due to both images being afflicted by opposite biases, such that their average largely compensates (see Fig. 5(b)).

**Fig. 6.**
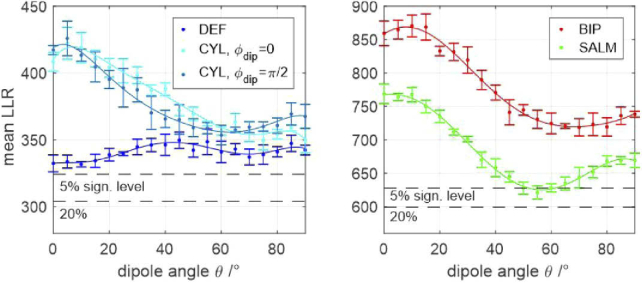
**Log-likelihood ratios of molecule image fits for fixed-dipole emission.** Left: Single-channel methods; Right: Dual-channel methods; The plots quantify each methods’ ability to identify fixed-dipole emission. Every data point represents an average LLR value over the entire z-range. Each errorbar marks the standard deviation of the respective z-averaged LLR value, calculated from 5 subsequent trials, assuming the signal and background values stated below. The black dashed lines mark the 5% and 20% significance thresholds. If the curves fall below these lines, fixed-dipole emission remains undetected by the localization algorithm, thus corrupting the data by introducing the biases shown in Fig. 5. Assumed parameters: UAF signal = 2000 photons, background = 100 photons per pixel.

Analogous to the case of aberrations discussed earlier, the methods’ capabilities for detecting errors caused by fixed-dipole emission are investigated by LLRs of simulated molecule images. The results are shown in Fig. 6. Each data point corresponds to the average LLR value of 23 simulated molecule images with equally spread z-positions within the entire z-range. The assumed values for signal and background level in each image are 2000 and 100, respectively. All methods are capable of identifying fixed dipole emitters at this signal and background level. The results further show that CYL and BIP have a higher probability of identifying fixed dipole emitters than DEF and SALM. Compared to the other methods, DEF performs particularly poor for z-dipole emission.

## 5. Localization errors caused by multiple emitter events

Another factor that influences localization performance is the blinking density of fluorophores. A higher density shortens the overall acquisition time but decreases the theoretically achievable localization precision. In recent years, various methods have been developed for the regime of dense emitters (see e.g. references in [58]). However, most SMLM algorithms still aim at localizing individual molecules, which provides the highest precision but demands a certain minimum separation between adjacent blink events. If a user-defined threshold distance is undercut, the event is classified as “multi-emitter event” and excluded from the analysis to maintain data fidelity. Problematic, however, are multi-emitter events which are not recognized as such, as they may lead to significant systematic errors in the parameter estimates. Both, the ability of a method to recognize multi-emitter events as well as its robustness to unrecognized ones are important quality criteria.

To investigate the impact of unrecognized multi-emitter events on the localization accuracy, we simulated the imaging of two closely spaced molecules at varying lateral separations and applied the single-emitter estimators for DEF, CYL, BIP and SALM. One molecule is always placed in the center of the FOV and the second one slightly laterally displaced. Two molecules at separations below a few camera pixels can hardly be distinguished by standard pre-selection algorithms, which are commonly based on detecting local maxima in a smoothed camera image. Therefore, we consider five lateral molecule separations ranging from 0 to 250 nm, at varying z-positions between 0 and 250 nm.

As DEF, BIP and SALM use rotationally symmetric PSFs with respect to the z-axis, the azimuthal position of the second emitter does not influence the result. Since CYL uses an asymmetric PSF, separations along the x- and y-axes (the symmetry axes of the astigmatic PSF) are individually investigated.

**Fig. 7.**
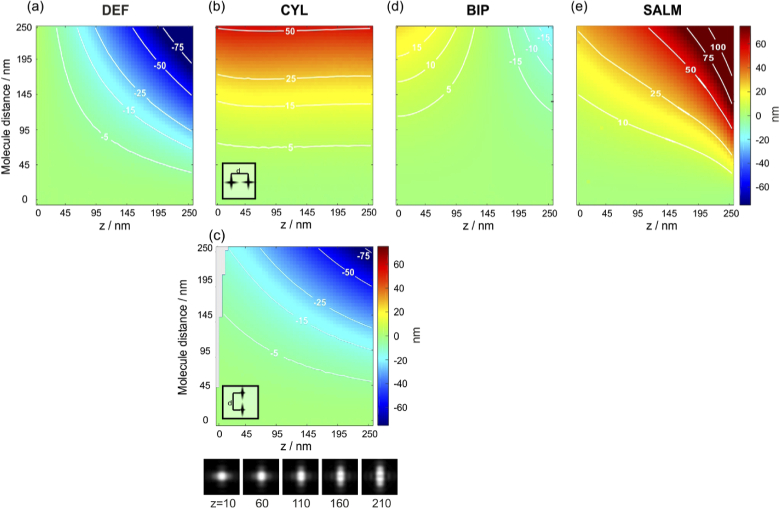
**Systematic errors of z-estimates caused by two close emitters**. The position estimators of DEF (a), CYL (b,c), BIP (d) and SALM (e) are applied to simulated images of close emitter pairs, which are laterally separated by 0 to 250 nm. The z-positions of the pairs are varied between from 0 to 250 nm. For CYL, the orientation of the emitter pair plays a role. The gray area on the left in (c) marks regions where the localization algorithm runs into its lower boundary (0 nm).

The simulation results are shown in Fig. 7. (a) shows the results for DEF, which generally underestimates the z-position at the presence of a second emitter. This is intuitively understandable, since a close pair of fluorophores resembles a more strongly defocused single molecule, which is interpreted as a lower z-position. This insight is also of relevance for the application of DEF to molecule tracking, where any image-smearing effects caused by fast diffusion in combination with long camera exposure times will likewise lead to a systematic underestimation of z-positions. (b,c) show the result for CYL, where we have to discriminate the cases of emitter pairs oriented along the x- and y-axes. If the pair is oriented along x (b), the z-position is overestimated, because the image of the pair resembles an ellipse of equal orientation, thus mimicking a single molecule at higher z-position. This becomes clear when considering the image table at the bottom, which shows CYL molecule images together with their z-positions. The bias is almost independent from the real z-position but gets amplified for larger pair separations, due to the elliptic shape becoming more pronounced. Conversely, when placing a second molecule along the y-axis (c), the algorithm tends to underestimate the molecule position, similar as for DEF. Shifting the second emitter in any direction between x and y results in an averaged deviation. If the second molecule is placed at $45^{\circ }$ to the x-axis, the deviation is minimal. (d) shows the results for BIP, which proves to be very robust to multi-emitter events. Finally, (e) shows the results for SALM.

As already mentioned, the robustness of a method to unnoticed multi-emitter events is an important characteristic. However, of likewise importance is a method’s ability to identify such events in the first place in order to remove them from the data. While fast pre-localization algorithms cannot distinguish two emitters which are closer than about an Airy disc, model-based sub-pixel estimators such as discussed here are able to detect them by quantifying the discrepancy of the molecule image to the best model fit, for instance using the LLR metric.

**Fig. 8.**
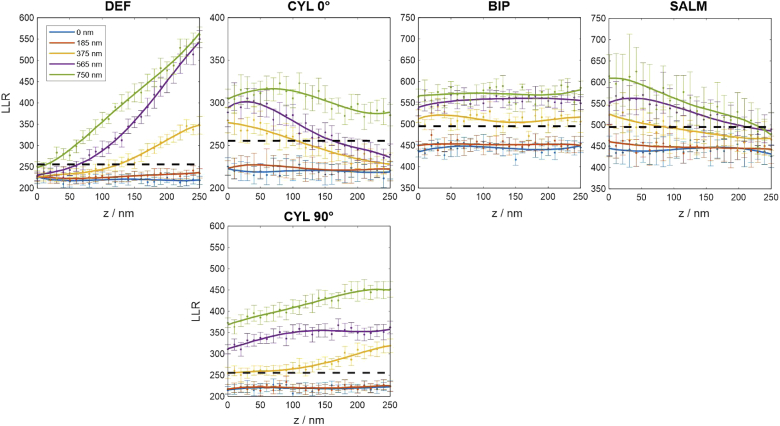
**Log-likelihood ratios of simulated images containing two emitters, for various lateral emitter distances**; Each datapoint is the average of 50 trials, assuming signal and background level as stated below. The black dashed line marks the 5% significance threshold. If the curves fall below this line, the double-blink event remains unrecognized by the localization algorithm, thus corrupting the data by introducing the biases shown in Fig. 7. Assumed parameters: UAF signal = 2000 photons, background = 100 photons per pixel.

Figure 8 shows LLR curves for all methods. The data indicates that defocused molecule images are difficult to separate, which appears plausible. These occur for DEF and SALM at very small, respectively large z-positions. There, both methods can only hardly identify emitter pairs with lateral separations smaller than about 500 nm, even for a relatively strict significance threshold of 5%. CYL suffers from the same problem if the z-position is large and the molecules are separated along the x-axis, where the PSF has its largest lateral extension. The information diversity provided by BIP, which always contains a rather in-focus image in one of the channels, renders it the best-performing method in this comparison.

## 6. Experimental results

### 6.1. Setup

**Fig. 9.**
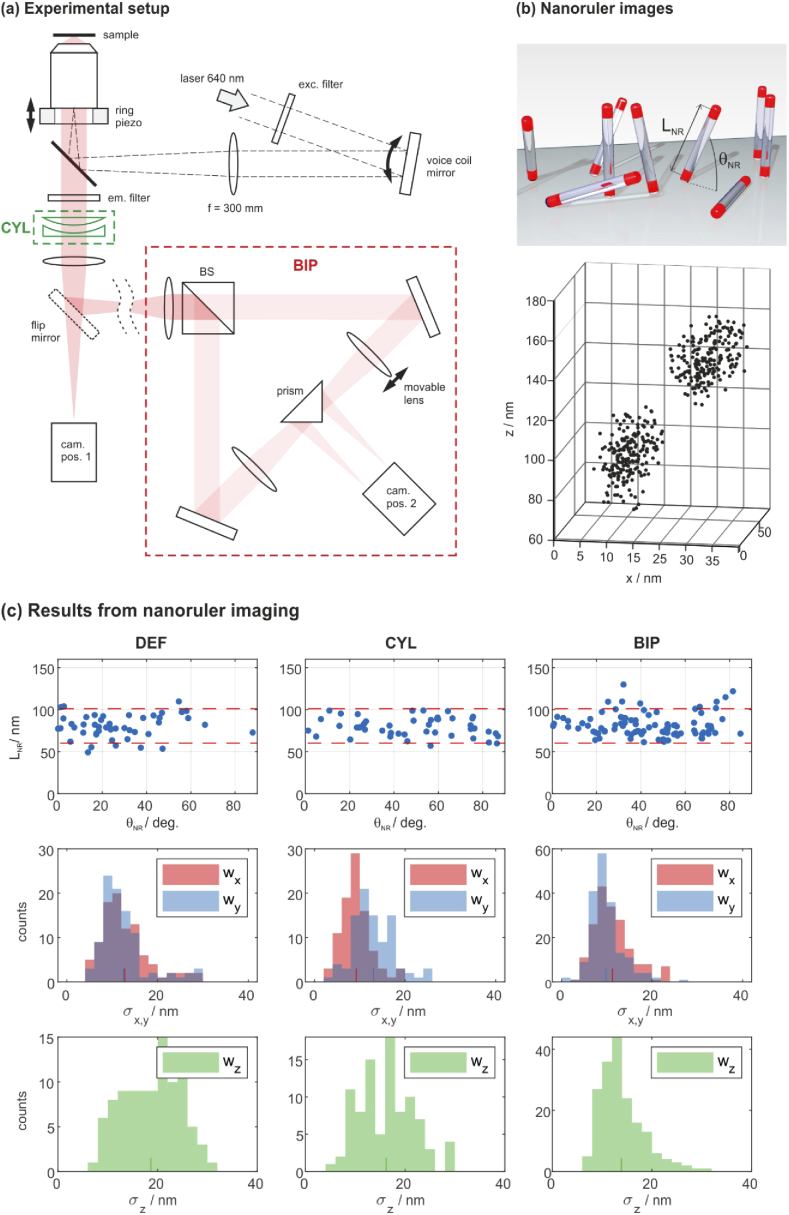
**Experimental results**; (a) Setup: the CYL and BIP modules are optional. (b) Nanoruler sample: the schematic structure of the sample is shown at the top. An exemplary image is shown below. Each dot marks a localization event. The z-axis falsely suggests a nanoruler position of approx. 80-150 nm above the coverslip. This error is due to a wrong assumption on Γ, which however only causes of global axial shift of the entire dataset. (c) Results from DNA PAINT imaging of DNA origami nanorulers: 1st row: distribution of lengths and angles; the red lines mark the manufacturers’ specifications. 2nd and 3rd rows: distribution of cluster sizes; the histograms show RMS deviations from the cluster centers along the x-, y- and z-directions. The respective mean values are indicated by short coloured marks on the horizontal axes.

The performances of DEF, CYL and BIP have been experimentally tested using a home-built microscope, which allows one to apply each of these methods. The setup is shown in Fig. 9(a). The objective lens is a TIRF lens from Olympus (APO N 60x Oil TIRF, NA 1.49). All further lenses in the detection path are microscope tube lenses from Nikon (focal length = 200 mm, Edmund Optics no.58-520). The following color filters are used: EX: ZET642/20×, EM: ZET532/640m-TRF, DICHR: ZT532/640rpc-UF2. A flip mirror allows for switching between single- and biplane modes and a cylindrical lens module can be inserted in front of the first tube lens for astigmatic imaging. The CYL module consists of two stacked cylindrical lenses with focal lengths of 1 m and −1 m. One lens can be rotated in order to set the desired astigmatism magnitude. At this position of the module within the optical train, a rotational angle of about 1-$2^{\circ }$ is sufficient to set $a_{6}$ to the ideal value of about 0.16 λ. The tube lens in one of the BIP arms can be translated by a micrometer stage in order to set the parameter Δ. Our axial microscope magnification is ${\left(60\cdot 200/180\right)}^{2}=4444$, which means that meeting the optimal value of Δ=510 nm requires to offset the lens position by 2 mm.

Precise defocus values Γ can be set with a z-piezo stage (PIFOC objective scanning system from Physik Instrumente). The camera is a Hamamatsu ORCA-Fusion Digital CMOS camera (C14440-20UP). The excitation laser from a 640 nm fiber coupled diode (Toptica iBeam smart) is deflected from a voice coil mirror in a conjugate image plane, which controls the laser’s angle of incidence on the specimen.

### 6.2. Data processing

As shown in our previous work [33], precise knowledge of the PSF is essential to meet highest precision and accuracy in the data analysis. Prior to each measurement, we thus record the 3D image of a fluorescent microbead (Gattaquant GATTA-Beads) that is directly attached to the coverslip, by taking several images at different piezo z-settings. Note that this 3D image is not identical to the PSF, because SAF-emission is equally present in all recorded z-slices of the bead image. However, the 3D image can be used to infer wavefront aberrations by applying a phase retrieval algorithm [59]. These aberrations are modelled in the objective pupil as a weighted sum of Zernike modes and are finally used to construct the SAF-aided PSF. The parameters $a_{6}$ and Δ used by the methods CYL and BIP are measured as well: $a_{6}$ is directly contained in the retrieved Zernike series and Δ can be estimated from the recorded 3D bead image stack by finding the axial distance of maximal intensities in both imaging channels. The parameter least well known is the defocus value Γ, because it’s accuracy depends on how well the experimenter is able focus onto the coverlip surface in order to define z=0. If done manually, errors on the order of 100 nm are common. Fortunately, systematic errors in Γ cause to a very good approximation merely an axial shift of the entire dataset, which is tolerable for most applications.

Of note, our approach does not require any further calibration steps as often done in 3D SMLM to account for the refractive index mismatch between immersion oil and buffer medium. It is further worth mentioning that the PSF characterization and data processing steps for single-channel methods are identical for any given PSF shape, including DEF and CYL. In BIP, the lateral molecule coordinates are separately estimated in the two images and averaged using CRLB-based weights as shown in Eq. (1). The remaining parameters (z, σ, β) are jointly estimated from both recorded images (see appendix 7.3 for further information).

### 6.3. Results from DNA PAINT

A suitable sample to test accuracy and precision are DNA origami nanorulers from Gattaquant (GATTA-PAINT 3D HiRes 80R Expert Line). These consist of modified DNA strands that form pillars which stand at various angles to the coverslip surface [60]. Both ends of each pillar carry an ssDNA binding site for base-pairing with a short ATTO655 imager strand (present in the same sample) to enable DNA PAINT imaging. The dye molecules at both ends are separated by 80.6(20.5) nm. A sketch of their basic structure is shown in Fig. 9(b). The sample comes already prepared for SMLM imaging via DNA PAINT.

The sample was recorded with an acquisition time of 50 ms per frame. For every imaging method, 10.000 frames have been collected using TIRF excitation at power densities of roughly 5 ${\mathrm{kW/cm}}^{2}$. Excitation under TIR angles inhibits fluorescence from molecules floating in the bulk and therefore reduces the background level. It is, however, not strictly required for the measurements.

The mean signal per molecule image was about 4.000 photons for all methods. The mean background level was around 140 photons per pixel. To obtain the necessary precision, images containing less than 500 photons were rejected. Events containing more than 20.000 photons were likewise sorted out, as such bright signals most likely originate from multi-emitter events. Further LLR-based filtering was performed, which removes events that significantly deviate from the PSF model. The filtering steps led to a 45% reduction of the DEF raw imaging data. The CYL data was reduced by 16 % and the BIP data by 23%.

The final data sets comprised ≈400.000 (DEF), ≈250.000 (CYL) and ≈200.000 (BIP) localizations, which were clustered according to their 3D position using a density based scan method [61]. A detailed description of the clustering approach is given in the appendix. Nanorulers were identified and their lengths $L_{NR}$ and inclusion angles to the cover slip surface $\theta_{NR}$ were determined. The data are shown in the first row of Fig. 9(c). Each blue spot marks a nanoruler and the red dashed lines bound the expected length range as stated by the sample manufacturer. The measured lengths are in excellent agreement with the expectation for each method. The numbers of identified nanorulers are 48 (DEF), 46 (CYL) and 91 (BIP). It can be assumed that these numbers correlate with a general prowess for localizing molecules close to the coverslip, which renders BIP as most suitable amongst the methods tested. The distribution of $\theta_{NR}$ may serve as a further quality criterion. According to the manufacturer, the angular distribution should be almost equal between ${\mathrm{20}}^{\circ }$ and ${\mathrm{90}}^{\circ }$, which matches the CYL and BIP data well. The DEF data shows a significant drop of recognized rulers at $\theta_{NR}>60^{\circ }$, indicating that the clustering algorithm often fails to resolve the two clusters of upright standing nanorulers. The measured cluster sizes, however, suggest that the axial localization precision of DEF should easily allow for resolving them. We therefore hold the DEF-related artefact discussed in section 5. responsible for this effect: Images showing both dye molecules of a nanoruler can be interpreted as a single dye molecule at an intermediate z-position. Only a few false localizations that fill the gap in between two clusters may already prevent their separation by the clustering algorithm.

The dimensions of each cluster reflect the respective localization precisions ($\sigma_{x},\sigma_{y},\sigma_{z}$) and are calculated as the RMS deviations of localizations from the cluster center. The cluster center is the mean 3D position of all contained localizations. The distributions of $\sigma_{x},\sigma_{y},\sigma_{z}$ are shown in the histograms of Fig. 9(c). The mean values ${\bar{\sigma }}_{x}/{\bar{\sigma }}_{y}/{\bar{\sigma }}_{z}$ represent the experimentally obtained precisions. They are marked with small vertical lines on the x-axes in the respective colors and further listed in Table 2, together with the theoretical precisions in brackets.

**Table 2.** **Measured localization precisions**. The CRLB-derived theoretical minimum values are stated in brackets. The numbers are in nanometers.

	${\bar{\sigma }}_{x}$	${\bar{\sigma }}_{y}$	${\bar{\sigma }}_{z}$
DEF	13 (13)	13 (11)	19 (18)
CYL	10 (8)	14 (11)	16 (16)
BIP	12 (9)	11 (8)	14 (13)

The experimentally obtained axial precisions are in very good agreement with the theory. The lateral precisions are slightly worse than predicted by the CRLB, which is most likely due to residual lateral drifts.

## 7. Summary and discussion

We have presented a detailed performance comparison of four methods for 3D SMLM in close proximity to the coverslip, where SAF effects play a role: defocused imaging, cylindrical lens imaging, biplane imaging and SALM. The comparison includes numerical investigations on 3D localization precisions and errors introduced by aberrations, fixed dipole emission and double-emitter events.

We have identified optimal imaging parameters for each of the methods, which provide best axial localization precision. These parameters are the defocus setting Γ, which exists all four methods, an astigmatism parameter $a_{6}$ for CYL and a defocus difference Δ for BIP.

Three of the methods (DEF, CYL, BIP) have been experimentally realized and tested using DNA PAINT on DNA origami nanoruler samples. The experimentally obtained precisions are in good accordance with the predicted values. Our approach of careful PSF calibration in combination with maximum likelihood estimation has been proven to avoid systematic errors in z-position estimates for all tested methods.

Our results indicate that biplane imaging (BIP) provides the best performance. This may be somewhat unexpected, given the fact that it has been developed to maximize the axial working range of a microscope rather than for near-field enhanced measurements. BIP has the best 3D localization precision as well as highest robustness to aberrations, dipole effects and multi-emitter events. These properties can be explained by the high informational diversity provided by the two imaging channels. However, these benefits come at the cost of a higher experimental complexity and higher computational efforts in data processing.

Amongst the single-channel methods, cylindrical lens imaging (CYL) performs best. It has a slightly better 3D precision than defocused imaging (DEF), a higher robustness to spherical aberrations (which are of particular importance) as well as a better ability to identify them from molecule images. It is also easier for CYL to reveal errors induced by fixed-dipole emitters.

SALM shows the lowest axial localization precision in this comparison and a very high precision loss in the presence of even very small negative spherical aberrations (Fig. 3(b)). However, it is important to note that SALM and dSALM are the only methods amongst the tested which can be applied to seemingly featureless structures such as densely labelled membranes. There is no requirement for single molecule observations. Indeed, SALM and related setups have originally been proposed for applications beyond SMLM [62–64]. Compared to the other methods, SALM exhibits a similar robustness to localization biases introduced by fixed dipole emitters. This is notable, given the fact that the method has been designed to be sensitive to the UAF/SAF energy ratio, which is highly dependent on the dipole orientation.

We note that our initial search for optimal imaging methods included the entire first-order Zernike set into the parameter space. Our optimization algorithm allowed for up to two imaging channels with individual pupil phase engineering using Zernike modes up to the first spherical term $Z_{11}$. We further allowed the split ratio for the two-channel methods to vary. This initial search showed that a split ratio of 50:50 is practically optimal and that the inclusion of Zernike modes other than $Z_{6}$ creates no further benefit. We believe that it is unlikely that higher Zernike modes beyond the first order could be supportive, because their higher spatial frequency content leads to a larger spatial extent of the PSF, which is generally unfavourable in the presence of a nonzero background. Of further note is that our search did not include phase masks containing singularities such as the family of helical PSFs [12,13] as this would have increased the search space beyond our current computational abilities.

## Figures and Tables

**Fig. 10.**
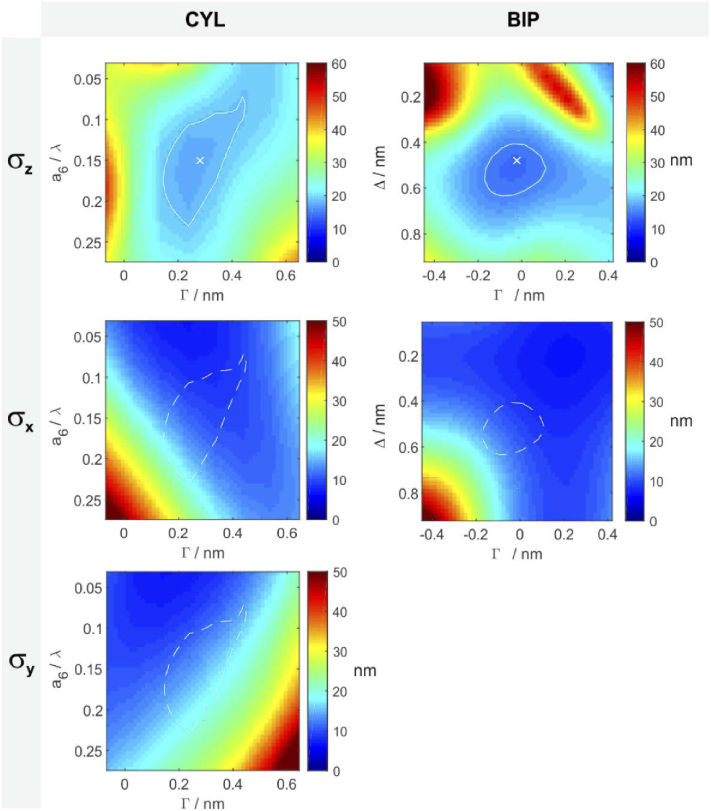
**Robustness of localization precisions with regard to parameter variations**; Boundary conditions: UAF signal = 2000 photons, background level = 100 photons per pixel, NA = 1.49.

**Fig. 11.**
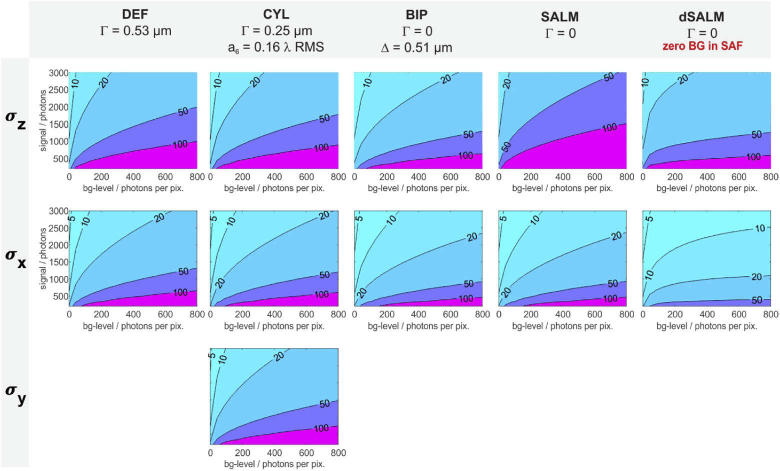
**Localization precisions for various signals and background levels**; the colors / contours mark average precisions (in nm) over the axial working range from 0 to $z=\lambda_{em}$/3 .

## Appendix

### 7.1. Robustness to parameter variations

The sensitivity of the localization performance to parameter variations, which are inevitable to some degree in experimental work, are visualized as color maps in Fig. 10. The color represents the mean localization precision over the entire z-range. Maps are only shown for the methods CYL and BIP. Note that the sensitivity of DEF to its single parameter Γ is contained in the CYL map along the horizontal line at $a_{6}=0$. The white crosses in the centers of the $\sigma_{z}$-maps mark the optimum and the white contour line a z-precision loss (i.e. a numerical increase of the best precision value) of 10%. Identical contours are also shown in the x and y precision maps. These results indicate that defocus variations on the order of 100 nm are uncritical regarding performance and a similar robustness in view of the cylindrical lens setting.

### 7.2. Precisions for different signals and background levels; dSALM

The contour plots shown in Fig. 11 show localization precisions over large ranges of signal and background levels. dSALM is included as well (fifth column). The performance of dSALM strongly depends on the background in the SAF channel, which is generally expected to be rather low, because only surface-near emitters produce SAF. The contour plot assumes a SAF-background level of zero, i.e., the optimal case. For dSALM, the background-axis (x-axis) thus shows the background of the UAF channel. dSALM shows its greatest advantage if the UAF background level is high, for instance due to fluorescence from distant emitters. This is explained by the fact that the SAF channel naturally does not contain this background. For the case of TIRF excitation, however, the difference between UAF and SAF background levels will be less pronounced and dSALM largely looses its advantage.

### 7.3. Data processing

For single channel imaging methods, the negative log-likelihood is calculated as follows:

(2) $$L(h,\theta ,I)\;=\;\sum_{k,l}^{N_{x},N_{y}}\mu_{k,l}(\theta )-I_{k,l}\;\log(\mu_{k,l}(\theta )),$$

with k,l representing the pixel indices and $N_{x},N_{y}$ the size of a single molecule image. $I_{k,l}$ describes the detected photons in pixel (k,l) and $\mu_{k,l}(\theta )$ the corresponding expectancy value. The latter depends on the signal σ, the background level β, the PSF $h_{k,l}$ as well as its position (x,y,z) within the image:

(3) $$\mu_{k,l}(\theta )\;=\;\beta +\sigma \;h_{k,l}(x,y,z),$$

with θ comprising all parameters to be estimated, i.e., θ=[x,y,z,σ,β].

For two-channel methods such as biplane imaging or SALM, the sum of two negative log-likelihood functions (one for each channel) is minimized for every molecule image:

(4) $$L_{tot}=L(h_{A},\theta_{A},I_{A})+L(h_{B},\theta_{B},I_{B}).$$

Note that here the subscripts A,B represent the respective imaging channel and not pixel indices.

Since each pair of images shows the same molecule, it’s five parameters should ideally be jointly estimated from both images. In this case, each image pair shares a common parameter vector $\theta =\theta_{A}=\theta_{B}$. This, however, requires very precise knowledge of the coordinate mapping between the two images, which can also slightly change over time due to drifts in the opto-mechanical elements. Alternatively, the lateral coordinates can be separately estimated in both images and then merged according to Eq. (1). Potential inaccuracies in the coordinate mapping would then only lead to minor systematic errors in the transverse positions, for instance a distortion of a few tens of nanometers across the entire image field. In this case, the parameter vectors are: $\theta_{A}=\left[x_{A},y_{A},z,R\sigma ,R\beta \right],\theta_{B}=\left[x_{B},y_{B},z,(1-R)\sigma ,(1-R)\beta \right]$, where R quantifies the intensity split ratio of the beam-splitter. R can significantly deviate from 0.5 (0.54 in our case) and should be measured beforehand.

For SALM and dSALM, the backgrounds should be estimated individually in both channels. The signals can be jointly estimated such as for biplane imaging, which, however, requires to normalize $h_{A},h_{B}$ such that method-dependent intrinsic signal differences between the channels (e.g. introduced by the SAF block in SALM) are accounted for.

### 7.4. Log-likelihood ratio test

The goodness of each fit can be assessed by computing the ratio of two negative likelihoods, respectively the difference between two negative log-likelihoods: The minimum value found by MLE and the negative log-likelihood, which results when the expectancy value $\mu_{k,l}$ is replaced by the actual measurement $I_{k,l}$:

(5) $$LLR=2\left(L(h,\hat{\theta },I)-\sum_{k,l}^{N_{x},N_{y}}\left(I_{k,l}-I_{k,l}\log(I_{k,l})\right)\right).$$

Here, $\hat{\theta }$ is the parameter vector that minimizes L. The factor of 2 ensures that the LLR values are approximately $\chi^{2}$-distributed, with the numbers of degrees of freedom determined as the number of pixels in a molecule image minus the number of parameters to be estimated. This allows us to conduct a log-likelihood ratio test for each fit: A fit is rejected if its LLR value exceeds a user-defined $\chi^{2}$ percentile. Equation 5 applies to the single-channel method but can be straightforwardly adapted for two channels.

### 7.5. Clustering

The clustering of the nanoruler data is performed in two steps based on density clustering [61]. Density-based clustering relies on two parameters: the minimum number of points $N_{min}$ in the neighborhood of a potential cluster member and the maximal allowed distance to those points $d_{max}$. A point belongs to a cluster if at least $N_{min}$ data points can be found at a maximal distance $d_{max}$ to the point. Adapting these two parameters according to the relevant cluster sizes allows for a controlled detection of clusters.

The first clustering step aims at separating individual nanorulers. In order to achieve that, the localization data is projected onto the x-y plane. Running the density-based clustering with $N_{min}=20$ and $d_{max}=30$ nm allows for localization of the nanorulers. The choice of the values is based on the minimal desired cluster size to allow for statistical analysis and on the maximal expected lateral distance between two clusters belonging to one nanoruler.

The next step is to identify the two parts of each nanoruler. To do this the clustering is repeated in three dimensions with $N_{min}=8$ and $d_{max}=20$ nm. The second clustering is performed separately for each cluster found in the previous step. The result is accepted as nanoruler if the algorithm finds two sub-clusters. Clusters in which only one or more than two sub-clusters are found are discarded. In a final manual step, clusters of supposed nanorulers which visually don’t appear separated are likewise discarded. The resulting pairs of sub-clusters are further analyzed by calculating their mean positions, cluster extensions, interspacings and orientations.
